# Surgeons' Preference Regarding Port Site Closure and Incidence of Port Site Hernias in Bariatric Surgery: A Retrospective Multicentre Study Across Peshawar, Pakistan

**DOI:** 10.7759/cureus.80982

**Published:** 2025-03-22

**Authors:** Muhammad Zareen, Saeed Sarwar, Zia Ullah, Abubaker Khattak, Rehan Saeed

**Affiliations:** 1 General Surgery, Khyber Teaching Hospital, Peshawar, PAK; 2 General Surgery, Khyber Medical College, Peshawar, PAK

**Keywords:** bariatric surgery complications, fascial closure, laparoscopy, port-site hernia, post-op complication

## Abstract

Introduction

Laparoscopic bariatric surgery is gaining popularity among the people of Pakistan because of increasing obesity and awareness about minimal access surgery. However, bariatric surgery is associated with certain potential complications. Port site hernias (PSHs) are one such complication and can be symptomatic or asymptomatic. The purpose of our study was to find the incidence of PSHs in our patients and the preference of operating surgeons regarding port site closure techniques.

Materials and methods

This study was conducted retrospectively by reviewing patients' records, history and examination with imaging, if necessary. Six hundred and fifty patients were included in the study who were operated between 2019 and 2024 across different centres in Peshawar. Data was collected about patients' age, gender, any comorbidities, type of surgery, surgeon preference for the closure technique and incidence of PSHs. Data was analysed using IBM SPSS Statistics for Windows, Version 26 (Released 2019; IBM Corp., Armonk, New York, United States).

Results

The incidence of PSHs in our patients was 1.5% (10 cases in 650). Three of the hernias occurred at a 10mm camera port site while others occurred at a 12mm left-side port site. Six of the patients were hypertensive and two were diabetic. Of the procedures performed, 202 were laparoscopic sleeve gastrectomy, 185 were laparoscopic roux-en-y gastric bypass and 263 were laparoscopic one anastomosis gastric bypass. All the port sites were closed using simple closure of skin incision only.

Conclusion

The incidence of symptomatic PSHs is low in our patients. The incidence of PSHs increases with the increase in the size of the port and comorbidities of patients. Simple skin closure was preferred by surgeons as it can reduce operative time, postop pain, surgical site infection and financial burden. However, further studies are needed for an ideal closing technique for port sites and to assess whether such closure decreases the incidence of PSHs.

## Introduction

Obesity is an increasing problem in large parts of the world, with rising prevalence in the Pakistani population. Bariatric surgery has emerged as a highly effective treatment for obesity and its associated comorbidities by affecting food intake, modulating gut hormone secretion, metabolic signalling pathways, and adipose tissue dysfunction [1] and recently gained popularity among the public in Pakistan due to minimally invasive techniques. While laparoscopic procedures have revolutionized the field, there are potential complications associated with bariatric surgery. Port site hernias (PSHs) are one such complication [2]. PSHs occur when a weakness develops in the abdominal wall at the site of trocar insertion, with protrusion of intra-abdominal contents through the defect. In addition to obesity, the use of large-sized trocars remains a risk factor for developing PSHs [3]. PSHs can cause significant discomfort and pain or may remain asymptomatic. Several closure techniques exist, including simple closure, subcuticular closure, and fascial closure [4-6]. Surgeon preference significantly influences the chosen closure method, with some surgeons preferring fascial closure while others do not. This study aims to investigate the preference of surgeons regarding port site closure and find the prevalence of PSHs among the local population.

## Materials and methods

Study design

This was a retrospective multicentre study conducted at Khyber Teaching Hospital Peshawar, Hayatabad Medical Complex Peshawar and Khyber Medical Centre Peshawar. Six hundred and fifty patients were included in this study.

Inclusion criteria

Both genders were included in this study. Patients who had undergone surgery in one of the three centres were included.

Exclusion criteria

Patients whose surgery was performed within six months from starting of this study were excluded.

Follow-up period

The minimum follow-up period was six months from the date of surgery.

Data selection

Data were collected retrospectively from electronic medical records, operative notes, and postoperative follow-up records including telephonic contact with patients who underwent primary bariatric surgery (Roux-en-Y gastric bypass, sleeve gastrectomy, one anastomosis gastric bypass). Patients who underwent revisional bariatric surgery with incomplete medical records or with prior abdominal surgeries were excluded. Data were collected about the patient's age, gender, body mass index (BMI), comorbidities (diabetes, hypertension), type of bariatric surgery, surgeon preference regarding port site closure and occurrence of PSHs.

PSHs were diagnosed based on the clinical presentation, examination and ultrasonography. All of the hernias were confirmed by ultrasonography.

Data analysis

Data were analysed using IBM SPSS Statistics for Windows, Version 26 (Released 2019; IBM Corp., Armonk, New York, United States). Descriptive statistics were used to summarize patient demographics, surgical details, and postoperative outcomes. Frequencies and percentages were calculated for categorical variables and means and standard deviations were calculated for continuous variables.

The study protocol was approved by the Institutional Review Board of Khyber Medical College Peshawar.

## Results

The study reports a prevalence of PSHs of 1.5%, with 10 cases identified in 650 patients who underwent laparoscopic bariatric surgery. Among the patient population, the mean BMI was 46.88 ± 6.77 and the mean age of the patients was 41.5 ± 9.77.

**Figure 1:**
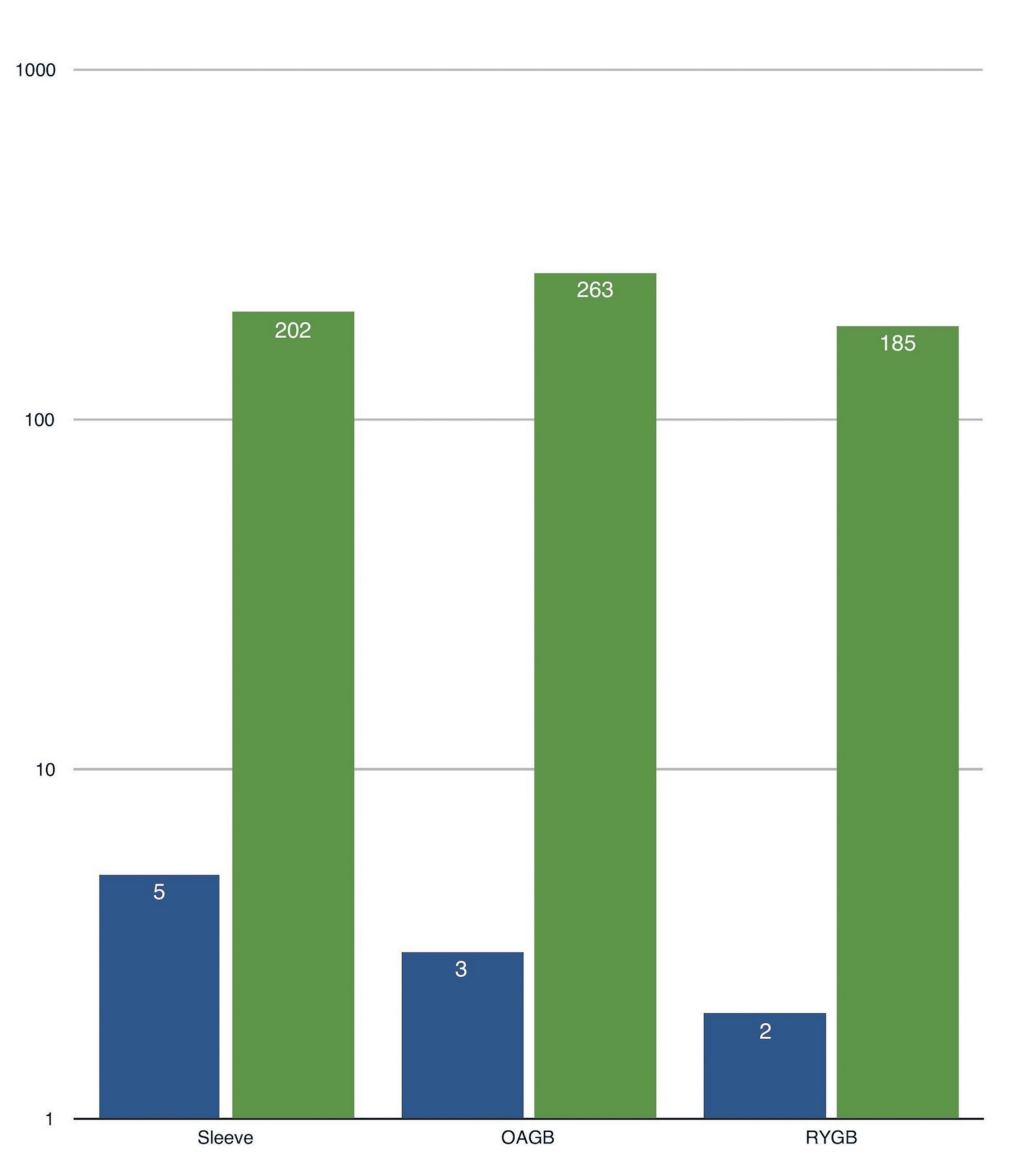
Surgery type and hernia number Green column: Total number of procedures performed. Blue column: Number of hernias in the specific procedure. All figures are shown in numbers (N) OAGB: One anastomosis gastric bypass; RYGB: Roux-en-Y gastric bypass

Of the 650 surgeries performed, the breakdown by procedure type was as follows: 202 patients underwent laparoscopic sleeve gastrectomy, 185 patients underwent laparoscopic Roux-en-Y gastric bypass, and 263 patients underwent laparoscopic one anastomosis gastric bypass. The specific procedure type performed may be of interest as it may have an impact on the likelihood of developing complications such as PSHs, though the study does not differentiate the rates of hernia occurrence by the procedure type. These numbers are illustrated in Figure 1, which provides a visual representation of the distribution of procedures.

**Figure 2:**
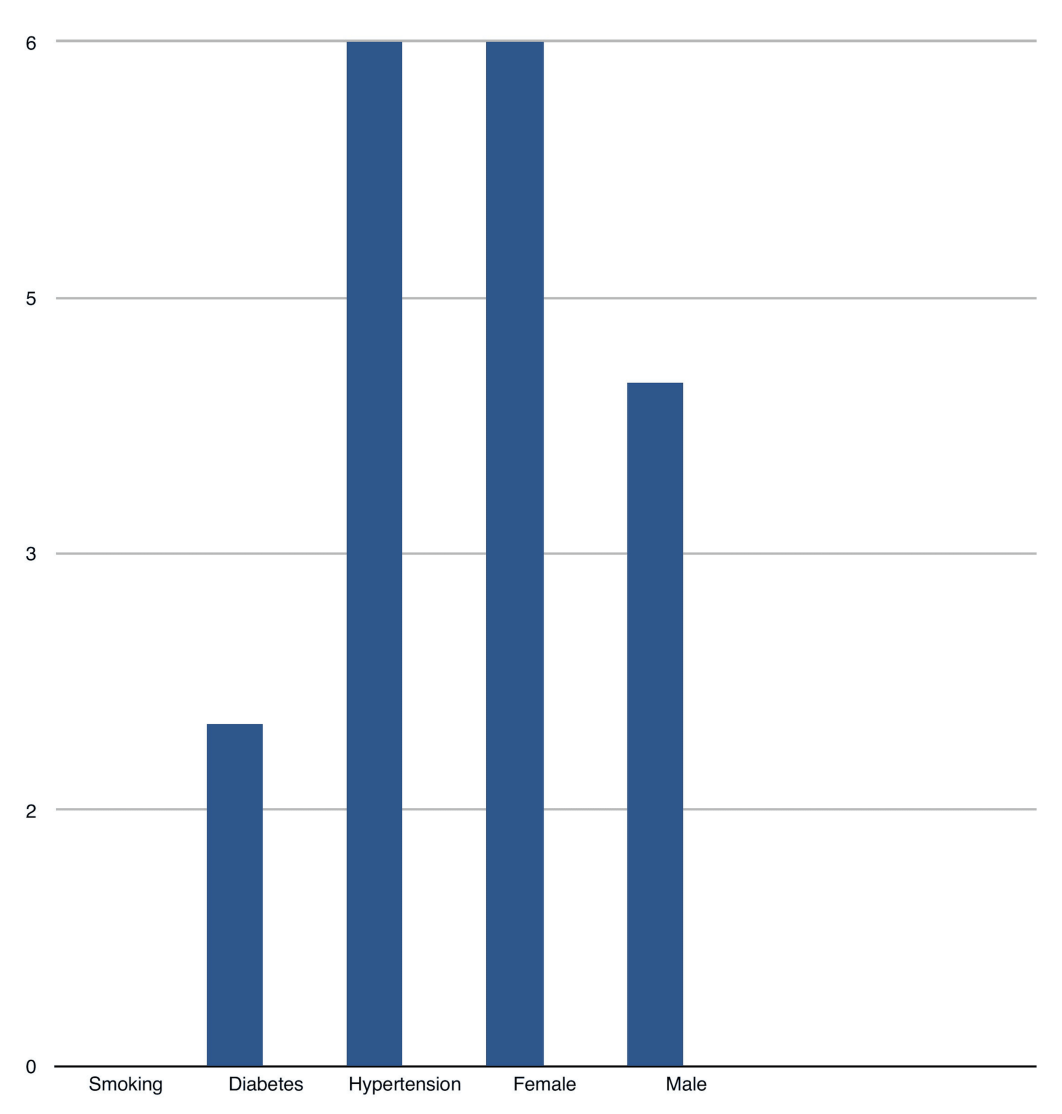
Clinical characteristics of patients Figures are shown in numbers (N).

Figure 2 details the clinical characteristics of the 10 patients who developed PSHs. Of the 10 patients, six were female and four were male, suggesting a possible gender difference in the incidence of this complication. Regarding comorbidities, six patients had hypertension, two patients were diabetic, while the remaining patients had no significant comorbidities. The presence of hypertension and diabetes could suggest that these factors may contribute to the risk of developing complications after surgery, though the study does not fully explore this relationship.

**Figure 3:**
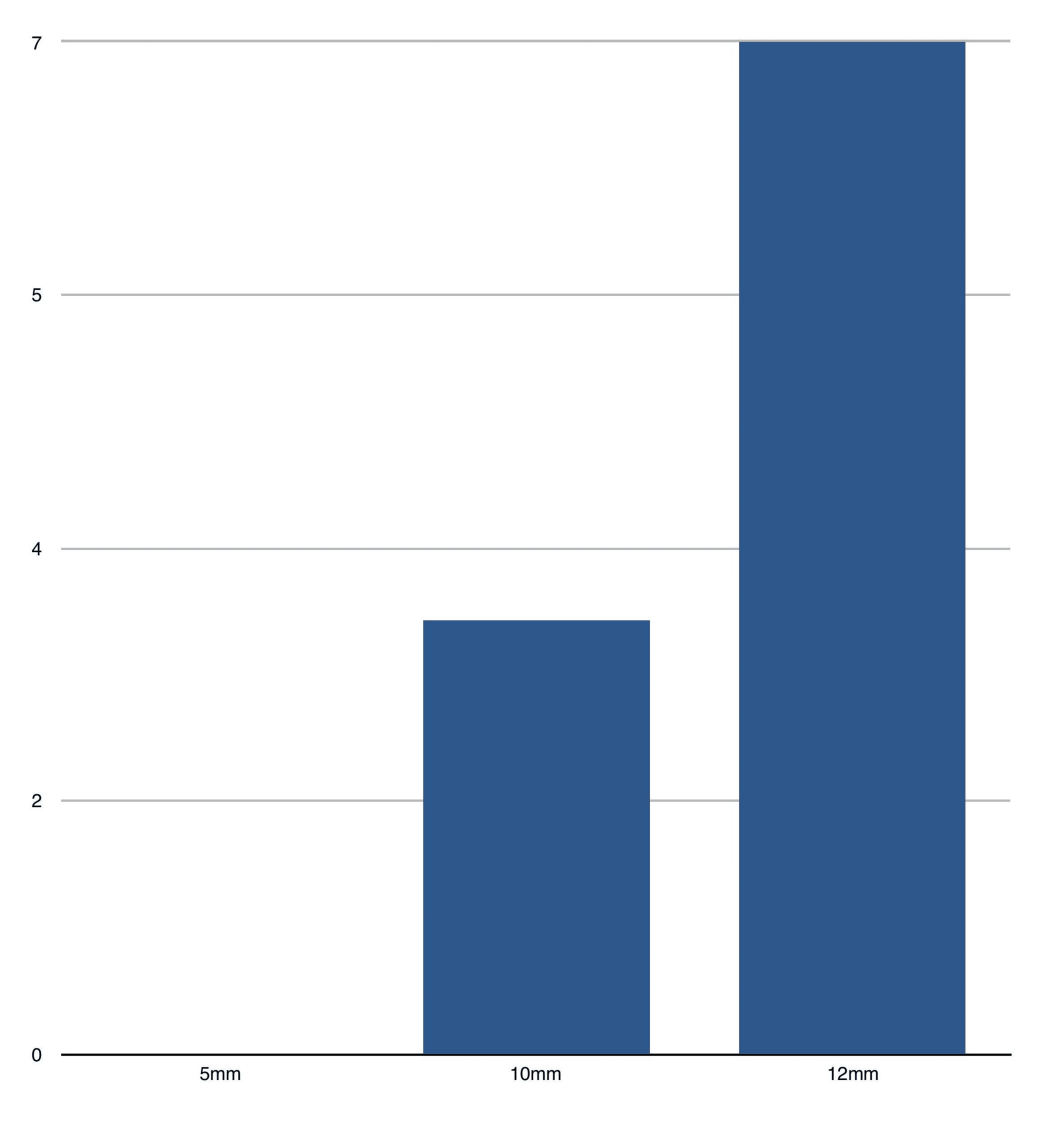
Port size and number of hernias All figures are shown in numbers ( N)

Regarding the location and size of the port sites, three of the 10 PSHs occurred at the 10mm camera port, while seven occurred at the 12mm midclavicular ports (as shown in Figure 3). These findings are consistent with a previous study [7] that suggests that larger port sizes, particularly those greater than 10mm, are associated with an increased risk of PSH formation. 

Four of the patients underwent surgical repair of their hernias due to increasing pain at the hernia site. The study does not elaborate on the type of repair performed, but this may involve simple closure or more advanced techniques depending on the size and symptoms. All surgeons involved in the study preferred simple closure of the port site, which involved closing only the skin incision.

## Discussion

PSHs are incisional hernias occurring at the port insertion site used during laparoscopic procedures. PSHs can be asymptomatic or cause symptoms related to strangulation or obstruction of abdominal contents protruding through the abdominal wall defect. Risk factors specific to PSHs are unclear. These hernias can be diagnosed using different imaging modalities like ultrasonography or computed tomography. The incidence of PSHs in our study is 1.5%.

Ahlqvist et al. reported a 21.7% incidence of trocar site hernia [8]. The reason for a stark difference between our study and this study is that we included only symptomatic patients in our study while the aforementioned study included asymptomatic patients as well. Furthermore, computed tomography was used to detect PSH in all of the patients included in the study.

Karampinis et al. included 365 patients in their study who were operated between 2009 and 2018 [9]. All the patients were subjected to ultrasonography for detection of PSH. They reported a 34% incidence of PSH. Similar to Ahlqvist et al., this study also subjected all the patients to imaging regardless of whether they were symptomatic or asymptomatic. Furthermore, their sample size was comparatively smaller than our sample size.

Coblijn et al. conducted their study on 1524 patients who were operated between 2006 and 2013 [10]. They reported an incidence of 0.5% for trocar site hernias. Their study is similar to ours in the aspect that only symptomatic patients were further investigated with imaging studies. Our incidence rate and theirs are comparable due to this reason.

Various techniques of port site closure are mentioned in the literature which includes simple skin closure and placement of mesh in the port sites [11]. Skin-only closure was preferred by surgeons in our study as it reduces both the operative time and financial burden of mesh on patients. In addition, there are concerns of more postoperative pain and surgical site infection in patients who had undergone fascial closure of port sites [12].

PSHs are more commonly associated with sleeve gastrectomy and large-sized ports, particularly greater than 10 mm. Sleeve gastrectomy was performed more in our patients which could contribute to the increased incidence of PSHs in our study.

Most PSHs are asymptomatic; however, the incidence of these asymptomatic hernias may be underestimated. This could be because patients are not routinely monitored for such issues or because the hernias do not cause overt symptoms that prompt further investigation. Therefore, longer follow-up periods and the use of imaging studies may be necessary to detect these hernias and understand their true prevalence. This would help ensure that potential issues are identified before they evolve into more serious complications that could affect the patient’s recovery or long-term health.

The incidence of PSHs in postop bariatric patients is high when patients are subjected to imaging investigations. However, symptomatic PSH incidence is comparatively low. How many of these asymptomatic cases will become symptomatic is a question to be answered and whether all patients need to go through imaging studies for PSH is a matter of controversy. Most of the surgeons do a simple closure of the port site as fascial closure may increase postoperative pain and its benefit in decreasing PSH rate formation is unclear. Further research is needed to address the aforementioned issues. Limitations of this study include a short follow-up period, a smaller sample size, and non-inclusion of other closure methods. The follow-up time in our study is variable as this was a retrospective study. A longer follow-up period is required to assess how many of the asymptomatic hernias become symptomatic. We did not include any other closure method as none of the surgeons included in our study used them.

In summary, the study highlights a relatively low prevalence of PSHs (1.5%) in a large cohort of patients undergoing laparoscopic bariatric surgery, with certain clinical characteristics such as hypertension and diabetes possibly influencing the development of this complication. The choice of port size and site, particularly the 12 mm midclavicular ports, may also play a role in the incidence of PSHs.

## Conclusions

Based on the preferences of bariatric surgeons, skin-only closure is the preferred method for closing port sites after bariatric surgery. Additionally, the incidence of symptomatic PSHs, which would require surgical intervention, is relatively low among bariatric surgery patients. This indicates that, while hernias can occur, they are not a frequent cause of clinical concern requiring further surgery.
